# Footprint coverage comparison between knotted and knotless techniques in a single-row rotator cuff repair: biomechanical analysis

**DOI:** 10.1186/s12891-019-2479-2

**Published:** 2019-03-25

**Authors:** Jair Simmer Filho, Andreas Voss, Leo Pauzenberger, Corey R. Dwyer, Elifho Obopilwe, Mark P. Cote, Augustus D. Mazzocca, Felix Dyrna

**Affiliations:** 1 Department of Orthopaedic Sports Medicine, Technical University of Munich, Munich, Germany; 2 Department of Orthopaedic Surgery, University of Connecticut, Farmington, CT USA; 3 Department of Orthopaedic Surgery, Hospital Estadual de Urgência e Emergência (HEUE), Vitória, Espírito Santo Brasil; 4 Department of Trauma Surgery, University Hospital Regensburg, Regensburg, Germany; 5 Vienna Shoulder & Sports Clinic, Vienna, Austria

**Keywords:** Rotator cuff, Cuff repair, Single-row, Biomechanics

## Abstract

**Abstract:**

**Background:**

The objective of this biomechanical study is to compare two variations of single-row knotless techniques (Knotless repair and Rip-stop Knotless repair) against a single-row double-loaded anchor (DL) repair, focused on evaluating contact pressure and contact area amongst three different single-row techniques for rotator cuff repairs.

**Methods:**

A total of 24 fresh frozen human shoulders were tested. Specimens were randomly assigned into one of the three single-row (SR) repair groups: A Knotted single-row double-loaded anchor (DL) repair, a Knotless (K) repair, or a Knotless Rip-Stop (KRS) repair. The footprint was measured after complete detachment of the supraspinatus tendon from the greater tuberosity, introducing pressure sensors between bony footprint and detached rotator cuff, and finally reconstructing it. All specimens were mounted onto a servohydraulic test system to analyze contact variables at 0° and 30° of abduction with 0 N, 30 N and 50 N of tension.

**Results:**

Groups did not differ significantly in their footprint sizes: DL group 359.75 ± 58.37 mm^2^, K group 386.5 ± 102.13 mm^2^, KRS group 415.87 ± 93.80 mm^2^ (*p* = 0.84); nor in bone mineral density: DL group 0.25 ± 0.14 g/cm^2^, K group 0.32 ± 0.19 g/cm^2^, KRS group 0.32 ± 0.13 g/cm^2^, (*p* = 0.75) or average age. The highest mean pressurized contact area measured for the K repair was 248.1 ± 50.9 mm^2^, which equals a reconstruction of 67.1 ± 19.3% at 0° abduction and a 50 N supraspinatus load. This reconstructed area was significantly greater compared with the DL repair 152.8 ± 73.1 mm^2^, reconstructing 42.0 ± 18.5% on average when under the same conditions (*p* = 0.04). The mean contact pressure did not significantly differ amongst groups (*p* = 1.0): DL group 30.8 ± 17.4 psi, K group 30.9 ± 17.4 psi and KRS group 30.0 ± 10.9 psi. Neither the 30° abduction angle nor the supraspinatus load had a significant influence on the contact pressure in our study.

**Conclusion:**

Both single-row knotless techniques resulted in significantly higher footprint reconstruction, providing larger contact area and a more uniform pressure distribution when compared with the single-row Knotted techniques. The mean contact pressure did not differ among groups significantly. These knotless techniques may be an alternative if the surgeon decides to perform a single-row rotator cuff repair.

**Level of evidence:**

Basic Science Study, Biomechanics.

## Background

The goals of rotator cuff repair are to achieve high initial fixation strength, minimize gap formation, maintain mechanical stability under cyclic loading in order to optimize the biology for the tendon to bone healing process [1, 2]. Ultimately, a successful repair should lead to the elimination of pain, improved strength, and an increase range of motion [1, 3]. Therefore, attention has been directed to improve biomechanical parameters of repair constructs by the modification of repair techniques, number of surgical implants, and invention of innovative materials [4, 5]. Appropriate use of those techniques is highly dependent on the surgeon’s experience and ability to manage an arthroscopic cuff repair [6].

Prior studies showed that increasing tendon loads would increase compression and frictional forces in addition to significantly increasing contact area and pressure. This was seen for both the transosseous equivalent (TOE) and single-row (SR) repairs [7]. While Double-Row (DR) repair is a widely used option offering biomechanical advantages, SR repairs are frequently used to treat small tears, in situ repair for partial tear, and also due to surgeon preference or cost issues [4, 5, 8, 9, 10–12]. Further discussions on the disadvantage of knotted compared with knotless fixation are still ongoing. Not only because it may increase operative time, but also because there is considerable variation in knot strength between surgeons [13]. Single-Row knotless repairs may be less technical demanding and can reduce operative time when compared to knotted repairs. In addition, it may have similar load to failure strength [11, 14]. On the other hand, the way that SR knotless fixation provides coverage of the footprint is not well investigated. Therefore, possible advantages regarding contact pressure and contact area are yet to be quantified.

The objective of this biomechanical study is to compare two variations of SR knotless repairs against a SR Knotted repair, focused on evaluating contact pressure and contact area amongst three different single-row techniques for rotator cuff repairs.

It was hypothesized that the knotless repair would result in significant higher contact area and lower contact peak pressure. Based on the two different techniques and philosophies of a knotted single row reconstruction compared to a laterally tensioned knotless technique. Number of anchors was equally to focus on the influence of the fixation techniques. The study was designed to investigate which repair performs biomechanically best under varying tension loading and different abduction angles.

## Methods

A biomechanical study was performed to compare two variations of single-row knotless (Knotless repair and Knotless Rip-Stop repair) against a Double-Loaded knotted repair, focused on evaluating the contact pressure and the pressurized contact area amongst these three different single-row techniques for rotator cuff repairs. Pressurized footprint is defined as the area that is not only covered but acutely loaded during repair testing and therefore represents the tendon repair more accurate than simply outlining the repair and additionally is less favorable for subjective bias and error. All specimens were obtained from Medcure Inc. (Portland, OR). The study was reported via Human Research Determination Form to the institutional review board (IRB) of the University of Connecticut and it was documented that no IRB approval was required (as de-identified specimen do not constitute human subjects research).

### Specimens preparation

A total of 24 fresh frozen human shoulders with a mean age of 67.2 years ranging from 50 to70 years, including 12 female and 12 male specimens were tested. Every shoulder was checked for macroscopic evidence of rotator cuff pathology and significant osteoarthritis, and exchanged if present. After thawed out for 24 h at room temperature, a bone mineral density evaluation via micro-CT (Lunar DXE, Madison, WI) was taken to assess bone quality at the site of suture anchor insertion at the greater tuberosity in a consistent manner of a 1x1cm area. All specimens were prepared by removing all superficial soft tissues followed by disarticulation at the glenohumeral joint to isolate the supraspinatus muscle and its tendinous insertion for better access and visualization. The individual muscles of the rotator cuff were dissected free from the joint capsule. The humeri were cut 15 cm distal from the greater tuberosity and potted in a 1.5-in.-diameter polyvinyl chloride pipe with plaster of Paris. The rotator cuff tendon was sharply dissected from its insertion on the greater tuberosity and was left in continuity with the individualized supraspinatus muscle. The bony footprint was then marked and measured using a MicroScribe digitizer (Immersion, San Jose, CA) to trace the outline of the attachment [3, 15]. The proximal end of the supraspinatus muscle was sutured to a polyester tape, using No. 5 non-absorbable suture (FiberWire, Arthrex Inc., Naples, FL) with an interlocking whipstitch in order to apply various tendon loads [3, 16]. A physiological saline solution was used to keep specimens moist during all phases of dissection, preparation, and testing.

### Repair techniques

Prior to the repair, the footprint area was macroscopically cleaned and prepared. The shoulder specimens were randomly divided into one of the three repair groups. Eight specimens were assigned for each repair technique. Utilizing a pen and a digital caliper, the distance from the lateral tendon edge was kept equivalent for all suture passages through the tendon during the repair in all specimens. Every repair construct consisted of two anchors performed by a single surgeon (JSF).

### Double-loaded repair (DL repair)

**Fig. 1:**
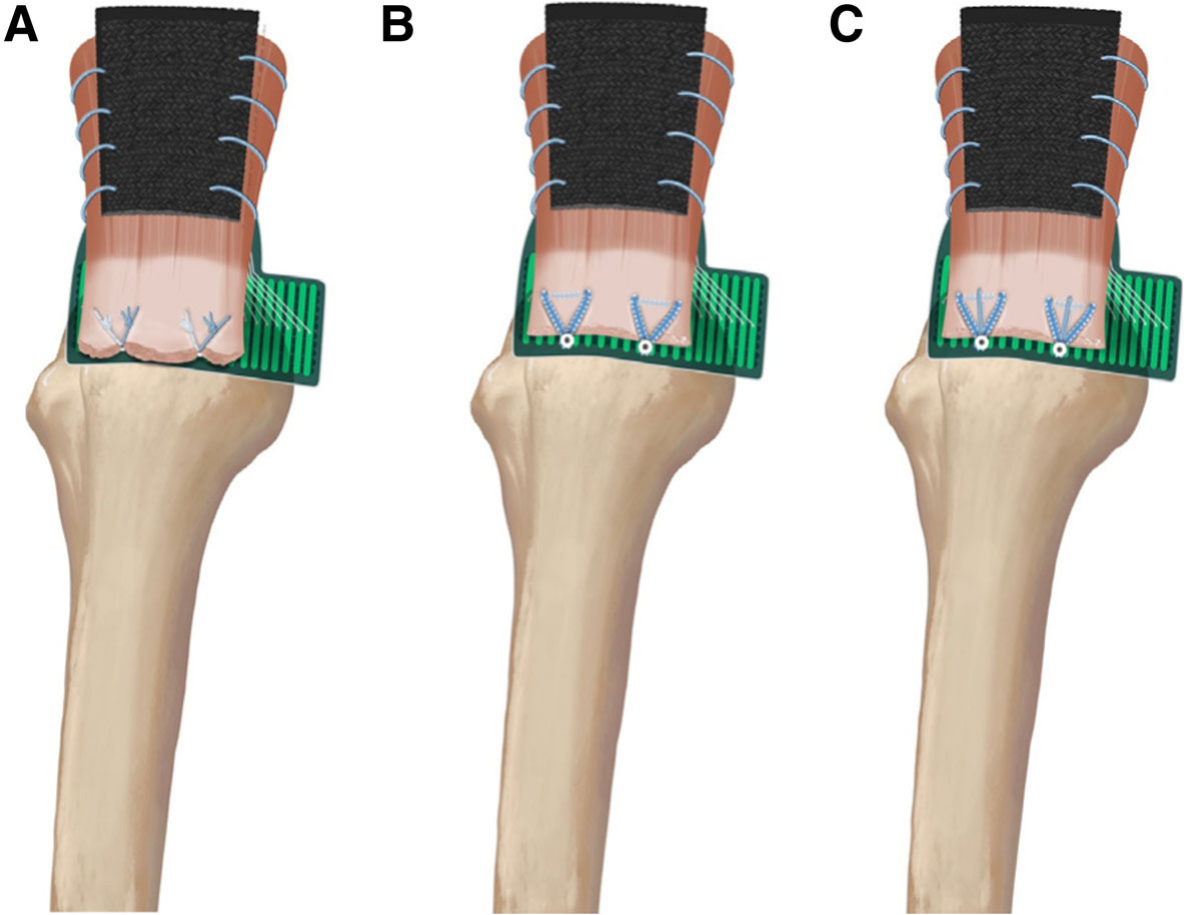
Illustration of the repairs with the TekScan sensor under the tendon in a left shoulder model. **a** Knotted Double-Loaded repair (DL repair). **b** Knotless repair (K repair). ***c*** Knotless Rip-Stop repair (KRS repair)

Starting 5 mm posterior to the bicipital groove, two holes were punched as far lateral as possible while still remaining on top of the footprint, thus maximizing the potential tendon contact area on the bony insertion. Each anchor was placed close to the lateral edge, 15.0 mm apart in the anterior to posterior direction. Two double-loaded anchors (4.5-mm Bio-Corkscrew FT® Arthrex Inc., Naples, FL) were used, and they were double loaded with #2 non-absorbable suture (FiberWire®). Simple suture configurations were used for this Knotted technique. The suture was passed 10.0 mm apart from one another for each given anchor, and 10.0 mm medial to the lateral edge of the simulated tear, with 5.0 mm separating both repair systems. All knots were tied with a Samsung Medical Center (SMC) sliding knot [17] followed by alternating 3 simple half-hitches, for a total of 4 throws using a knot-pusher (Fig. 1a).

### Knotless repair (K repair)

Two inverted mattress stitches with a 2 mm suture tape (Fibertape®; Arthrex Inc) sutures were passed 10.0 mm apart from one another and 10.0 mm medial to the lateral edge of the simulated tear, with 5.0 mm separating both repair systems. Starting 5 mm posterior to the bicipital groove, two lateral holes were made close to the lateral edge of the greater tuberosity and centered 15.0 mm apart. Two knotless anchors (4.75-mm Bio-Composite SwiveLock®; Arthrex) were used to fix the tape down into the tuberosity holes, after the surgeon tensioned it laterally over the tendon edge (Fig. 1b).

### Knotless rip-stop repair (KRS repair)

Two inverted mattress sutures with 2-mm suture tape (Fibertape®; Arthrex Inc) were passed 10.0 mm medial to the lateral edge of the simulated tear, approximately 10.0 mm apart. After that, a cinch suture (FiberLink®; Arthrex) was passed just medial to the inverted mattress suture tape. This step with the cinch suture was repeated in same fashion for the second anchor. Two lateral holes were punched in the same manner as for the Groups A and B. After the surgeon tensioned it laterally over the tendon edge, two knotless anchors (4.75-mm Bio-Composite SwiveLock®; Arthrex) were used to fix the suture tape and the cinch suture down into the tuberosity holes (Fig. 1c).

### Pressure sensor preparation

Pressurized contact area and contact pressure were measured using a Tekscan model 4205 sensor (Tekscan Inc., South Boston, MA). Tekscan sensors have the ability to continuously collect data points in real time. The matrix dimensions for the sensor we used was 41.9 mm by 45.7 mm. This working area surpasses the average footprint area, thereby allowing us to incorporate the sensor into the repair without losing sensitivity. The reliability and accuracy of the Tekscan sensors on a curved surface, such as the greater tuberosity, were ensured in previously published studies [3, 18]. Before every test, the sensor was precalibrated to a force and pressure consistent with previous rotator cuff repair studies [3, 7]. To incorporate the sensor, in all groups, two 4.0 mm holes were created by use of a sharpened leather punch into the Tekscan sensor, 12.0 mm apart on the lateral side. Those holes were made in order to introduce the anchors and sutures through the sensor to allow for interposition between the footprint and the cuff during all repairs. The sensors were sealed between 2 layers of clear tape to prevent moisture from entering and causing delamination of the sensor. During the repairs, the Tekscan sensor was placed between the supraspinatus tendon and the greater tuberosity footprint.

After the tendon was finally fixed, we determined the footprint area coverage by compressing the outline of the reconstructed area on the sensor. This marked the region of interest for further analysis. All contact variables were measured at 0° and 30° of abduction with 0 N, 30 N and 50 N of supraspinatus load, respectively.

### Biomechanical testing

**Fig. 2:**
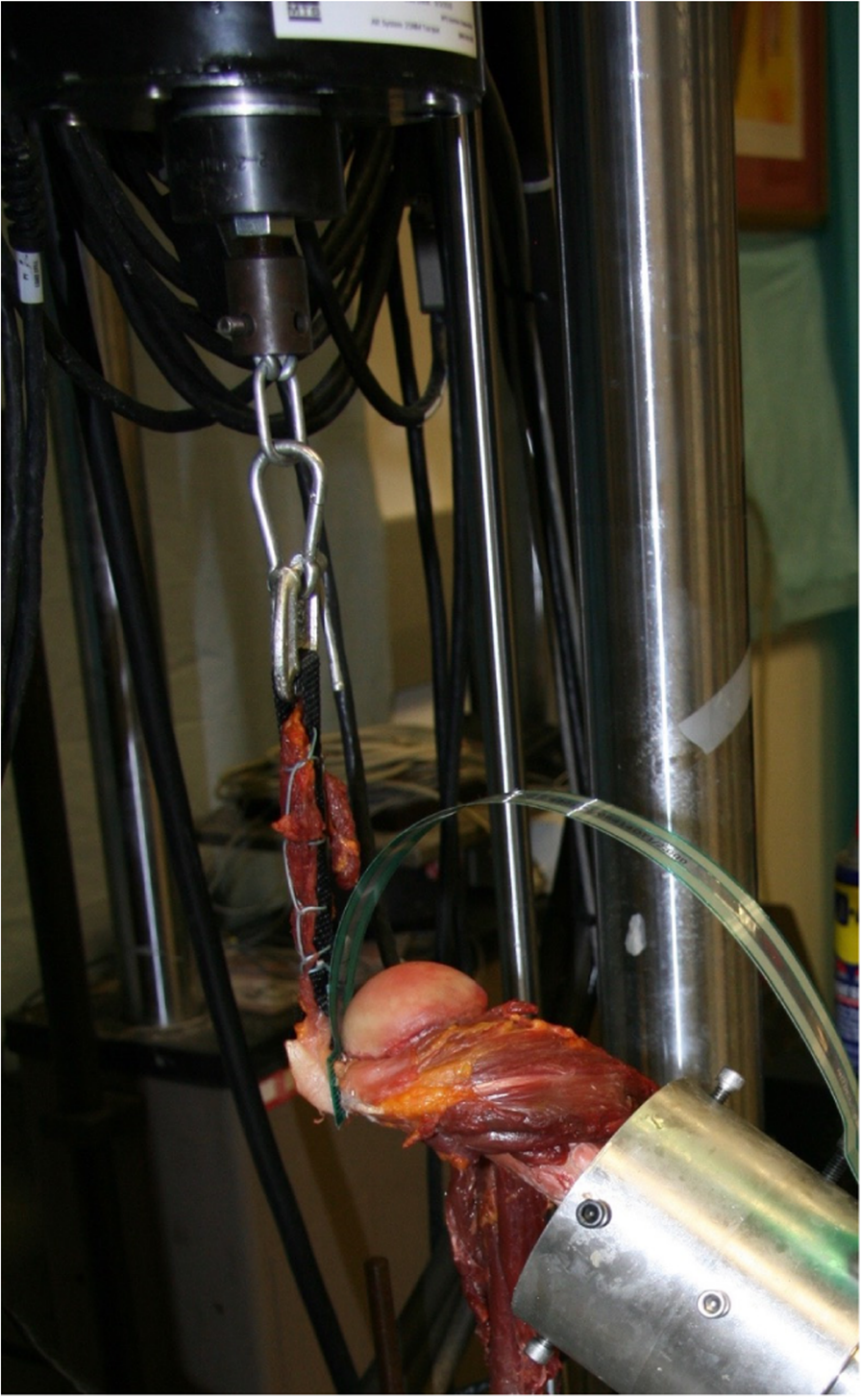
Right shoulder specimen with the TekScan sensor under the repaired tendon in the MTS machine at 30° of abduction and under supraspinatus load

Biomechanical setup and testing was performed in accordance with previously published protocols [3, 19]. The potted cadaveric specimens were then mounted on a custom shoulder platform and inserted into the mechanical testing system (MTS, Eden Prairie, MN). The humeri were centered in a cylinder attached to a jig that allowed free motion in the X-Y-Z planes to adjust for rotation and abduction angles. A goniometer was used to ensure neutral humeral rotation and 0° of abduction. After fixation within the MTS machine, the humeri were locked in neutral rotation by aligning the supraspinatus footprint dimension (anterior-to-posterior) perpendicular to the loading vector [20]. The loading vector line of action was defined by a No. 5 FiberWire® whip-stitched interlocking suture and a polyester tape aligned with the center of the muscle and parallel to the baseplate. The straps were locked to the actuator of the system to transfer loads. (Fig. 2) To evaluate the effect of increasing supraspinatus loads onto the footprint, contact pressure and contact area measurements were recorded at time zero after repair and with the following supraspinatus loads: 0 N, 30 N and 50 N. Measurements were taken in neutral humeral rotation at 0° and 30° of glenohumeral abduction for all repairs. For the unloaded measurements, a 10-s time frame was recorded directly after the repair. In order to capture the values for the loaded state, each specimen was tested following the listed protocol. First, preloading with a constant load of 30 N for 5 min was performed, to act as a pre-condition. This was followed by a force-controlled ramped loading up to 50 N and held for 30 s, recording contact pressure and area. Then a step down to 30 N for more 30 s recording contact pressure and area was carried out followed by unloading. Altogether, this simulates a physiological load that may be experienced postoperatively, as has been described in previous studies [21, 22]. The relative low load applied (50 N as a maximal simulation) was used as it best simulates the in vivo early postoperative load situation.

### Statistical analysis

There is no predicate for determining a relevant difference in footprint coverage. A difference in coverage of 10% between the repaired groups with an assumed standard deviation of 5 to 6% equates to an effect size of 1.75. A sample of 8 shoulders per group provides 80% power to detect a 10% difference in footprint coverage at an alpha level of 0.05. A Monte Carlo simulation of 1000 ANOVAs with an estimated 5.5% standard deviation among the groups resulted in 87% power to detect a 10% difference in coverage.

Descriptive statistics to characterize the study groups were calculated using mean and standard deviation. Differences between the groups were analyzed with a one-way ANOVA. When statistically significant, pairwise differences between the repair groups were analyzed with independent *t*-tests along with a Bonferroni adjustment. The alpha level for all analysis was set at 0.05. All statistical analyses were performed using Stata 12 (StataCorp. 2011. *Stata Statistical Software: Release 12*. College Station, TX: StataCorp LP).

## Results

Groups did not differ significantly in their native footprint size: DL repair 359.75 ± 58.37 mm^2^, K repair 386.5 ± 102.13 mm^2^ and KRS repair 415.87 ± 93.80 mm^2^ (*p* = 0.84). Nor did they differ in bone mineral density: DL repair 0.25 ± 0.14 g/cm^2^, K repair 0.32 ± 0.19 g/cm^2^ and KRS repair 0.32 ± 0.13 g/cm^2^ (*p* = 0.75) or in average age. The results are summarized in Table 1. There was no significant difference in the contact area amongst the three groups without a supraspinatus load (*p* = 0.87). However, when the supraspinatus was loaded, there was a progressive change in the percentages of footprint coverage (contact area) with statistically significant differences between DL repair and K repair (*p* = 0.03), regardless of load or abduction angle. Further, no statistical difference was observed between K repair and KRS repair (*p* = 0.59).

**Table 1:** Results for footprint contact, area, contact pressure and percent of reconstructed area

	0 N		30 N		50 N	
	0°	30°	0°	30°	0°	30°
Foot print contact area (mm^2)						
** Group DL**	66,3 ± 43,0	76,3 ± 40,0	135,1 ± 63,4	125,1 ± 56,4	152,8 ± 73,1	137,9 ± 70,9
** Group K**	77,5 ± 47,4	73,9 ± 40,8	230,4 ± 50,5	202,4 ± 62,9	248,1 ± 50,9	226,3 ± 72,4
** roup KRS**	70,8 ± 49,7	92,4 ± 33,3	198,3 ± 64,4	182,8 ± 59,8	222,1 ± 44,7	206,9 ± 66,3
T-Test DL vs K	0,6	0,9	0,0	0,0	0,0	0,0
T-Test DL vs KRS	0,8	0,4	0,1	0,1	0,0	0,1
T-Test K vs KRS	0,8	0,3	0,3	0,5	0,3	0,6
Foot print contact area %)						
** Group DL (A)**	17,7 ± 10,2	20,4 ± 8,8	37,1 ± 16,3	34,2 ± 14,4	42,0 ± 18,5	37,6 ± 18,2
** Group K**	20,0 ± 10,6	20,2 ± 13,1	62,5 ± 19,3	54,7 ± 20,8	67,1 ± 19,3	60,9 ± 21,9
** Group KRS**	16,8 ± 10,0	22,8 ± 8,0	50,9 ± 21,6	47,0 ± 20,5	56,4 ± 18,4	52,8 ± 21,2
T-Test DL vs K	0,7	1,0	0,0	0,0	0,0	0,0
T-Test DL vs KRS	0,9	0,6	0,2	0,2	0,1	0,1
T-Test K vs KRS	0,5	0,6	0,3	0,5	0,3	0,5
Foot print contact pressure						
** Group DL**	30,8 ± 17,4	33,4 ± 14,8	32,9 ± 12,8	33,9 ± 15,8	33,1 ± 12,1	34,8 ± 14,9
** Group K**	30,9 ± 17,4	31,1 ± 20,4	31,6 ± 13,10	31,7 ± 14,5	30,9 ± 10,9	31,9 ± 14,0
** Group KRS**	30,0 ± 10,9	29,6 ± 13,5	30,9 ± 7,5	29,5 ± 8,9	29,0 ± 8,2	29,2 ± 9,6
T-Test DL vs K	1,0	0,8	0,8	0,8	0,7	0,7
T-Test DL vs KRS	0,9	0,6	0,7	0,5	0,5	0,4
T-Test K vs KRS	0,9	0,9	0,9	0,7	0,7	0,7

The highest mean pressurized contact area was measured for the K repair 248.1 ± 50.9 mm^2^, at 0° abduction and a 50 N load, which equals a reconstruction of 67.1 ± 19.3%. This contact area was significantly higher when compared with the DL repair 152.8 ± 73.1 mm^2^, which only reconstructed 42.0 ± 18.5% on average under the same conditions (*p* = 0.04). The addition of a medial reinforcement stitch in KRS repair did not result in a further increase of contact area (222.1 ± 44.7 mm^2^) or of native footprint area (56.4 ± 18.4%) (*p* = 0.41). Increasing the abduction angle from 0° to 30° did not significantly decreased contact area. Similar results were measured when a decreased supraspinatus load (30 N) was applied (Table 1).

**Fig. 3:**
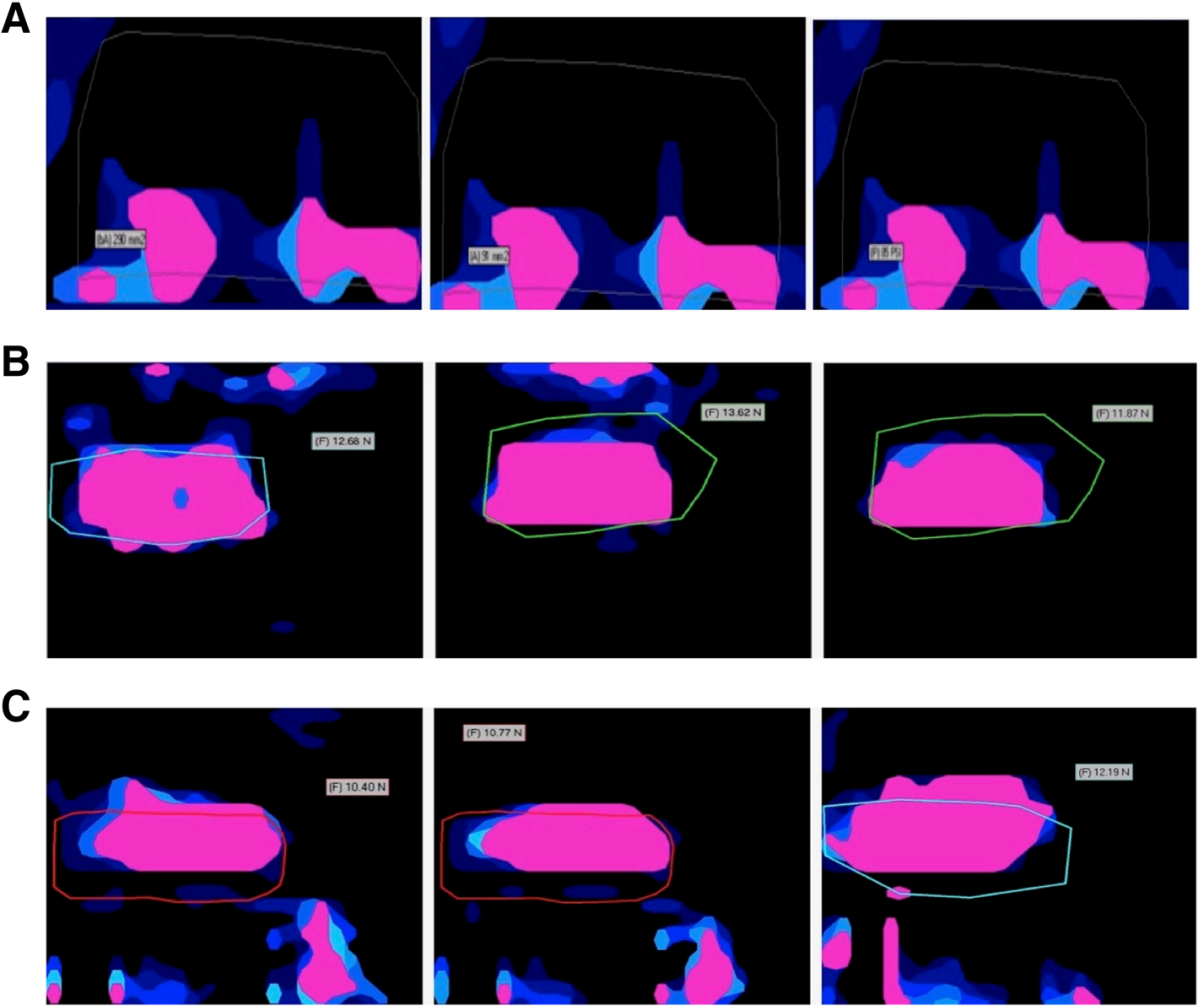
Pressure distribution and coverage footprint area. The brighter colored zones indicate a greater force than the darker areas. These images demonstrate the disturibution of force rather than the quantity of force. It is noted that K repair and KRS repair generate a more uniform distribution within the footprint area, which is outlined. The represented pictures demonstrated the condition (30 N 0° / 50 N 0° / 30 N 30° from left to right). **a** Knotted Double-Loaded repair (DL repair). **b** knotless repair (K repair). **c** knotless rip-stop repair (KRS repair)

The mean contact pressure did not differ significantly (*p* = 1.0): DL repair 30.8 ± 17.4 psi, K repair 30.9 ± 17.4 psi and KRS repair 30.0 ± 10.9 psi. Neither the 30° abduction angle nor the supraspinatus load had a significant influence on the contact pressure in our study. However, differences in load distribution were present, showing overall higher peak loads in the conventional SR surrounding the knots, with less uniform pressure dispersion compared to the knotless reconstructions without reaching significance. (Fig. 3a-c).

## Discussion

The current biomechanical study compared three different single-row rotator cuff reconstructions, focused on evaluating the contact pressure and the contact area amongst these different single-row techniques. Our most important findings were that under tension load both single-row knotless techniques (K repair and KRS repair) showed as expected, improved footprint coverage and a larger contact area when compared with single-row knotted technique (DL repair). Furthermore, both knotless techniques also showed more uniform pressure distribution patterns.

Potential advantages regarding footprint coverage and contact pressure of K repair or KRS repair had not been previously studied. An experimental study, comparing K repair and transosseous repairs for supraspinatus tears of 1.0 cm in cadaveric sheep shoulders, found no statistical significance in load to failure [14]. Further it has been shown that biomechanical properties of a single row repair with Triple Loaded Anchor versus KRS repair were similar without a statistical difference in load to failure or displacement during cycling loading [11]. However, none of these studies did evaluate footprint coverage and contact pressure of knotless techniques.

We found no significant differences in contact area or contact pressure when changing the abduction angle from 0° and 30° in each of the three repairs. Our study’s highest mean pressurized contact area was measured at 0° abduction and a 50 N supraspinatus load (K repair = 67.1 ± 19.3%). Park et al. investigated the influence of arm position in contact area, and they also found the highest contact area with arm position in 0° abduction with neutral rotation and under tendon load [23]. Although they found that arm abduction from 0° to 30° cause contact area decreases, it was not statistically significant. On the other hand, when the abduction angle was increased from 30° to 60°, it caused a significant decrease of the contact area after repair. These findings suggests that it might be important for the clinical practice to keep the immobilization of the shoulder with 30° or less of abduction during the post-operative period.

At 0° of abduction without supraspinatus load, there was no difference in the contact area amongst the three groups (DL repair 17.7 ± 10.1%, K repair 20.0 ± 10.6%, KSR repair 16.8 ± 10.0%). However, we could observe that tendon loads increased the contact area in all three reconstructions tested in the present study. Previously, it could be shown that increasing tendon loads may increases compression and frictional forces in addition to significantly increasing contact area and pressure [7]. This was seen for both the TOE and SR repairs [7].

Apreleva et al. [24] investigated the traditional double-loaded SR repair contact area in cadaveric human shoulders with a supraspinatus tear using a MicroScribe digitizer to outline the original tendon insertion and the reconstructed area after different techniques without an applied load. They found 67% footprint coverage after a knotted single-row DL repair, which was higher than our findings for the same technique (42.0 ± 18.5%). We attribute this difference to the used techniques. In the current study we used both a MicroScribe digitizer to outline the native tendon insertion and the Tekscan sensor to include only the pressurized footprint coverage overlapping the measures to calculate the percentage of coverage, whereas Apreleva et al. used only MicroScribe and therefore may overestimated the contact area after the repair. Similar to our results, Park et al.^12^ reported 38.3% in average of single-row DL repair of pressurized footprint contact area. The Tekscan sensor [3, 4, 23], used to measure the footprint contact characteristics, allowed the measurement of contact pressure in real time. It was also found that knotted single-row DL repair had a concentrated contact pressure, localized around the suture knots and anchor hole (Fig. 3a) but the mean contact pressure did not significantly vary amongst the groups (*p* = 1.0). Additionally, it has been observed that K repair and KRS repair showed a much more uniform distribution of contact pressure (Fig. 3a-c) without reaching significance, most likely due to not enough power and low sample count.

Given the same or better footprint coverage, knotless techniques may be advantageous as they limit the need to tie knots, which may have variable strength and depend on surgical skills. In addition, it can potentially be performed quicker, reducing surgical time. Hanypsiak et al. found considerable variation and inconsistencies in ultimate load to failure between knots tied by different surgeons and even between knots tied by the same surgeon on the same occasion [13]. The variation in knot tying has the potential to affect the integrity of arthroscopic repairs and suggests that arthroscopic surgeon should be aware of the challenges of arthroscopic knot tying and the possibility that the knots can be a limiting factor.

### Limitations

There are several limitations to this study. This is a cadaveric study with macroscopically intact supraspinatus tendons, individualized and isolated detached from the greater tuberosity, may not mimic chronic and degenerative changes as expected in vivo. The use of the Tekscan sensor is susceptible to artifacts because of crinkling as well as edge effects. Ideally, it should be calibrated using surface topography places on a flat surface. All repairs should be performed tension-free, which may not be the case in clinical practice. In addition, none of the repairs were loaded to failure or cyclic loaded. Although both contact pressure and contact area may be influenced by cyclic loading and load to failure test, they have not been performed because of the sandwiched Tekscan sensor interposed between the bone and the tendon limiting the biomechanical rational.

## Conclusions

Both single-row knotless techniques resulted in significantly higher footprint reconstruction, providing larger contact area and a more uniform pressure distribution when compared with the single-row Knotted techniques. The mean contact pressure did not differ among groups significantly. These knotless techniques may be an alternative if the surgeon decides to perform a single-row rotator cuff repair.

## Abbreviations

DL: Souble loaded
DR: Double row
IRB: Institutional review board
K: Knotless
KRS: Knotless Rip-Stop
MTS: Mechanical testing system
SMC: Samsung Medical Center
SR: Single row
TOE: Transosseous equivalent

## References

[1] Cole BJ, ElAttrache NS, Anbari A (2007). Arthroscopic rotator cuff repairs: an anatomic and biomechanical rationale for different suture-anchor repair configurations. Arthroscopy.

[2] Dyrna F, Buchmann S, Beitzel K, Imhoff AB (2016). Rotator cuff healing. Obere Extremität.

[3] Mazzocca AD, Bollier MJ, Ciminiello AM (2010). Biomechanical evaluation of arthroscopic rotator cuff repairs over time. Arthroscopy..

[4] Park JS, McGarry MH, Campbell ST (2015). The optimum tension for bridging sutures in transosseous-equivalent rotator cuff repair: a cadaveric biomechanical study. Am J Sports Med.

[5] Mascarenhas R, Chalmers PN, Sayegh ET (2014). Is double-row rotator cuff repair clinically superior to single-row rotator cuff repair: a systematic review of overlapping meta-analyses. Arthroscopy..

[6] Guttmann D, Graham RD, MacLennan MJ, Lubowitz JH (2005). Arthroscopic rotator cuff repair: the learning curve. Arthroscopy.

[7] Park MC, McGarry MH, Gunzenhauser RC, Benefiel MK, Park CJ, Lee TQ (2014). Does transosseous-equivalent rotator cuff repair biomechanically provide a “self-reinforcement” effect compared with single-row repair?. J Shoulder Elb Surg.

[8] Genuario JW, Donegan RP, Hamman D (2012). The cost-effectiveness of single-row compared with double-row arthroscopic rotator cuff repair. J Bone Joint Surg Am.

[9] Millett PJ, Warth RJ, Dornan GJ, Lee JT, Spiegl UJ (2014). Clinical and structural outcomes after arthroscopic single-row versus double-row rotator cuff repair: a systematic review and meta-analysis of level I randomized clinical trials. J Shoulder Elb Surg.

[10] Hein J, Reilly JM, Chae J, Maerz T, Anderson K (2015). Retear rates after arthroscopic single-row, double-row, and suture bridge rotator cuff repair at a minimum of 1 year of imaging follow-up: a systematic review. Arthroscopy..

[11] Noyes MP, Lederman E, Adams CR, Denard PJ. Triple-loaded suture anchors versus a knotless rip stop construct in a single-row rotator cuff repair model. Arthroscopy. 2018. 10.1016/j.arthro.2017.12.024. 10.1016/j.arthro.2017.12.024 29456064

[12] Vap AR, Mannava S, Katthagen JC, Horan MP, Fritz EM, Pogorzelski J, Millett PJ (2018). Five-year outcomes after arthroscopic repair of partial-thickness supraspinatus tears. Arthroscopy..

[13] Hanypsiak BT, DeLong JM, Simmons L, Lowe W, Burkhart S (2014). Knot strength varies widely among expert Arthroscopists. Am J Sports Med.

[14] Onay U, Akpınar S, Akgün RC, Balçık C, Tuncay IC (2013). Comparison of repair techniques in small and medium-sized rotator cuff tears in cadaveric sheep shoulders. Acta Orthop Traumatol Turc.

[15] Curtis AS, Burbank KM, Tierney JJ, Scheller AD, Curran AR (2006). The insertional footprint of the rotator cuff: an anatomic study. Arthroscopy.

[16] Mazzocca AD, Millett PJ, Guanche CA, Santangelo SA, Arciero RA (2005). Arthroscopic single-row versus double-row suture anchor rotator cuff repair. Am J Sports Med.

[17] Barber FA, Drew OR (2012). A biomechanical comparison of tendon-bone interface motion and cyclic loading between single-row, triple-loaded cuff repairs and double-row, suture-tape cuff repairs using biocomposite anchors. Arthroscopy..

[18] Park MC, Jun BJ, Park CJ, Ahmad CS, ElAttrache NS, Lee TQ (2007). The biomechanical effects of dynamic external rotation on rotator cuff repair compared to testing with the humerus fixed. Am J Sports Med.

[19] Beitzel K, Chowaniec DM, McCarthy MB (2012). Stability of double-row rotator cuff repair is not adversely affected by scaffold interposition between tendon and bone. Am J Sports Med.

[20] Kim SH, Ha KI, Kim JS (2001). Significance of the internal locking mechanism for loop security enhancement in the arthroscopic knot. Arthroscopy.

[21] Drewniak EI, Crisco JJ, Spenciner DB, Fleming BC (2007). Accuracy of circular contact area measurements with thin-film pressure sensors. J Biomech.

[22] Reilly P, Bull AMJ, Amis AA (2004). Passive tension and gap formation of rotator cuff repairs. J Shoulder Elb Surg.

[23] Park MC, Pirolo JM, Park CJ, Tibone JE, McGarry MH, Lee TQ (2009). The effect of abduction and rotation on footprint contact for single-row, double-row, and modified double-row rotator cuff repair techniques. Am J Sports Med.

[24] Apreleva M, Ozbaydar M, Fitzgibbons PG, Warner JJP (2002). Rotator cuff tears: the effect of the reconstruction method on three-dimensional repair site area. Arthroscopy.

